# Pathophysiological concepts and screening of cardiovascular disease in dialysis patients

**DOI:** 10.3389/fneph.2023.1198560

**Published:** 2023-09-29

**Authors:** Gift Echefu, Ifeoluwa Stowe, Semenawit Burka, Indranill Basu-Ray, Damodar Kumbala

**Affiliations:** ^1^ Division of Cardiovascular Medicine, The University of Tennessee Health Science Center, Memphis, TN, United States; ^2^ Department of Internal Medicine, Baton Rouge General Medical Center, Baton Rouge, LA, United States; ^3^ Department of Internal Medicine, University of Texas Rio Grande Valley, McAllen, TX, United States; ^4^ Department of Cardiology, Memphis Veterans Affairs (VA) Medical Center, Memphis, TN, United States; ^5^ Nephrology Division, Renal Associates of Baton Rouge, Baton Rouge, LA, United States

**Keywords:** chronic kidney disease, hemodialysis, left ventricular hypertrophy, uremic toxins, beta 2-microglobulin, galectin-3, suppression of tumorigenicity-2 (ST2)

## Abstract

Dialysis patients experience 10–20 times higher cardiovascular mortality than the general population. The high burden of both conventional and nontraditional risk factors attributable to loss of renal function can explain higher rates of cardiovascular disease (CVD) morbidity and death among dialysis patients. As renal function declines, uremic toxins accumulate in the blood and disrupt cell function, causing cardiovascular damage. Hemodialysis patients have many cardiovascular complications, including sudden cardiac death. Peritoneal dialysis puts dialysis patients with end-stage renal disease at increased risk of CVD complications and emergency hospitalization. The current standard of care in this population is based on observational data, which has a high potential for bias due to the paucity of dedicated randomized clinical trials. Furthermore, guidelines lack specific guidelines for these patients, often inferring them from non-dialysis patient trials. A crucial step in the prevention and treatment of CVD would be to gain better knowledge of the influence of these predisposing risk factors. This review highlights the current evidence regarding the influence of advanced chronic disease on the cardiovascular system in patients undergoing renal dialysis.

## 1 Introduction

Over 450,000 individuals are currently on maintenance dialysis in the United States (1). Despite recent advances in the dialysis process, these patients continue to experience high hospitalization rates, poor quality of life, and increased annual mortality above 20% of the general population. Chronic kidney disease (CKD) impacts the cardiovascular system tremendously and is regarded as an independent risk factor (1, 2). Survival rates for patients on dialysis are poor, at 40%, regardless of the mode of dialysis (3, 4). Cardiovascular disease mortality accounts for a significant proportion of all-cause mortality in this population (3, 4). A growing body of evidence endorses that CKD patients are more likely to die from cardiovascular disease-related adverse events than from events related to kidney failure (5, 6). It is estimated that around 80% of hemodialysis patients suffer from at least one form of cardiovascular disease at the time of dialysis initiation, stemming from preexisting cardiovascular risk factors in these patients (7–10). Among these conditions are left ventricular hypertrophy (29%–75%), congestive heart failure (20%–40%), coronary artery disease (22%–39%), atrial fibrillation (11%–27%) and valvular heart disease (24%) (10–17). Traditional and nontraditional risk factors, not limited to hypertension and hyperglycemia, altered bone mineral metabolism, endothelial dysfunction, volume overload, and uremic toxins contribute to the development of CVD in this population (18–22).

Creatinine-based estimated glomerular filtration rate and albuminuria are independent predictors of poor cardiovascular disease outcomes (23). In fact, microalbuminuria in the absence of overt renal dysfunction confers an increased risk of cardiovascular disease by two to fourfold (24). Go et al (25), in one of the most powered studies, evaluated the impact of kidney dysfunction on cardiovascular events, mortality, and hospitalization in an adult population of >1.1 million in northern California. Baseline GFR was estimated from the abbreviated Modification of Diet in Renal Disease (MDRD) equation (26). The authors used the modified National Kidney Foundation classification (27) of chronic kidney disease to longitudinally stratify eGFR among the study subjects. The mean follow-up period was 2.84 years. After adjusting for comorbidities, age, sex, ethnicity and socioeconomic status, the risk of cardiovascular events, mortality and hospitalization increased as the eGFR decreased below 60 ml per minute per 1.73 m2 of body-surface area (25). An important limitation to this study, which is not uncommon, is the exclusion of patients on dialysis and renal transplant recipients. This reiterates the current challenges in managing cardiovascular disease in dialysis and transplant recipients as several landmark and clinical practice changing investigations often exclude these patients. Furthermore, there is heterogeneity in the inherent predisposing factors, distribution, and types of cardiovascular disease manifestations in individual CKD patients. Therefore, the extent to which these traditional interventions can deliver clinical benefits in this population remains uncertain.

Studies comparing cardiovascular outcomes among patients on HD and those on peritoneal dialysis report higher mortality association with PD. The contributing mechanisms are unclear but proposed hypotheses include unpredictable and inadequate fluid removal and higher atherogenic lipid profile leading to higher risk CVD in PD (28–30). A comparison of 3468 HD patients with 933 PD patients in an observational study revealed that PD significantly increased the risk of all-cause mortality including, cardiovascular mortality.

The first part of this review focuses on contemporary evidence regarding the impact of advanced kidney disease on the cardiovascular system in patients undergoing renal dialysis, the predisposing traditional and nontraditional risk factors. The second part reviews the pathophysiologic drivers of several CV diseases and current screening tools and biomarkers.

## 2 Pathophysiological mechanisms of specific cardiovascular diseases in dialysis

### 2.1 Left ventricular hypertrophy

Left ventricular hypertrophy (LVH) in dialysis patients is a complex physiologic adaptive response to long-term increase in volume or pressure load (31). It is not an uncommon complication among pre-dialysis patients with CKD (32, 33). LVH is prevalent in up to 75% of CKD patients at initiation of dialysis (34). Concentric (from LV pressure overload) and eccentric (from volume overload) patterns have been reported in literature (11). Prominent predisposing factors comprise of anemia, hypertension, dialysis vintage, volume overload, interdialytic weight gain and bone-mineral metabolism (35–38). Anemia is an established reversible risk factor for LVH among patients on dialysis (37, 39, 40). Anemia in CKD is primarily due to insufficient endogenous erythropoietin production. Furthermore, iron deficiency develops from over consumption associated with accelerated erythropoiesis following exogenous EPO, gastrointestinal bleeding (from uremia and arteriovenous malformations) and unavoidable blood loss associated with HD access cannulation (32). There is a significant link between left ventricular mass (LV) and volume with the degree and duration of anemia in patients with end stage renal disease (ESRD) (31). A decrease in hemoglobin concentration as low as 1 g/dL has been linked to a heightened risk of LV dilatation, systolic dysfunction, heart failure, and mortality (37, 41–43). The interplay of hemodynamic and non-hemodynamic mechanism compensates for anemia in patients with ESRD (31). Enhanced erythropoiesis from increased endogenous erythropoietin production and increased tissue oxygen extraction mediated by 2,3-diphosphoglycerate concentrations are the main non-hemodynamic compensatory mechanisms in anemia (31). Hemodynamic compensatory mechanisms are mediated by increase in preload (via venoconstriction), positive inotropy and chronotropy (via sympathetic mediators) and reduced afterload, to maintain appropriate cardiac output (31). Vasodilatory mechanisms and reduced vascular resistance resulting from tissue hypoxia, increased nitric oxide activity and lower blood viscosity are instrumental to maintaining reduced after load. Increased venous return increases the preload and left ventricular filling, in turn increasing the left ventricular end diastolic and stroke volumes. Enhanced contractility is attributable to increased concentrations of catecholamine and other inotropic factors. Chronic hemoglobin concentration less than 10-11 g/dL has been implicated as the clinical threshold for anemia induced LVH (35). In non-exertional states and hemoglobin concentration >10 g/dL, the non-hemodynamic mechanisms alone sufficiently compensate for anemia without engaging hemodynamic factors (44, 45). In contrast, hemodynamic mechanisms become necessary when hemoglobin concentration falls below <10 g/dL or with exertion (44, 45). As anemia progresses in chronicity, the maladaptive phase ensues as these mechanisms become ultimately overwhelmed resulting in often irreversible cardiomyopathy and left ventricular hypertrophy (46). Additionally, in ESRD patients, sustained increase in cardiac output induces arterial remodeling (intima-media thickening) in the large vessels (aorta and carotids) and interstitial fibrosis contributing further to the maladaptive phase (31, 47, 48).

Hypertension is a known risk factor for LVH in non-dialysis patients (34, 36, 49). It is not unlikely that the aging ventricle is relatively sensitive to hypertrophic effect of systolic blood pressures (36). Increased afterload stemming from increase in peripheral resistance and impedance causes myocardial remodeling, thickening of myofibers and then concentric hypertrophy. This pathological form of LV remodeling is often in concert with interstitial fibrosis (50). A prospective study enrolled fifty-one ESRD patients with no evidence of LVH on baseline echocardiogram at the initiation of dialysis. Mean follow up duration was twenty-nine months. Cases had remarkably higher systolic blood pressure than controls (149.6 [SD, 13.4] mm Hg versus 137.1 [SD, 15.7] mm Hg; P = 0.0091). There was a positive correlation (r = 0.39; P 0.01) between systolic blood pressure and final left ventricular wall thickness (36). Overall, age and systolic blood pressure were independent predictors of development of LVH (36). A study showed a correlation between pre-dialysis systolic blood pressure between LV mass in hemodialysis patients. The authors propose that controlling pre-dialysis BP alongside post dialysis systolic BP is more impactful than maintaining post dialysis SBP in the normal range. This study enrolled very few subjects therefore these claims warrant higher powered studies to confirm its merits.

High levels of phosphorus are independently linked to increased LV mass in dialysis patients (51). The mechanism is still unclear, but studies report an association with vascular calcification as well as arterial stiffness leading to pressure overload on the left ventricle. Therefore, therapeutic interventions to lower phosphorus levels could improve cardiovascular outcomes (51). A multinational blinded randomized controlled trial demonstrated an association between dialysis vintage and left ventricular volume increase even after adjusting for age and gender (35). At baseline, patients with lower dialysis vintage had lower left ventricular volume increase (35).

FGF23 is markedly elevated in dialysis due to the feedback signal of intractable hyperphosphatemia and klotho insufficiency (52). Several studies support its association with LVH in uremic patients. There are theoretical pathogenic pathways for this relationship (52–55). In uremic patients, FGF23 is activated by the renin-angiotensin-aldosterone system which promotes pro-fibrotic interaction between cardiac myocytes and fibroblasts (56). Several other risk factors known to contribute to the pathogenesis of LVH in non-dialysis patients including diabetes have been explored in dialysis patients but was found to be noncontributory. This may be due to co-existence of other factors with more prominent influence such as hypertension, anemia, and volume overload in CKD patients (51).

### 2.2 Heart failure

Heart failure is a syndrome that comprises structural or functional cardiac dysfunction manifesting as failure of the heart to pump blood and maintain physiological circulation (51). HD patients are at risk of either diastolic or systolic dysfunction or both (51). The development of heart failure is mainly due to cardiomyopathy and ischemic disease. About 36% of ESRD patients requiring dialysis already exhibit clinical signs of HF at initiation of dialysis (57). CHF and chronic kidney disease share several overlapping risk factors and pathomechanisms, including renin–angiotensin–aldosterone system activation. ESRD patients with HF have poor survival facing as high as 25-35% mortality risk (11, 58–60). Hemodialysis is a well-documented independent cardiovascular stressor that can precipitate repetitive HD-induced myocardial stunning, resulting in LV dysfunction, myocardial hibernation, as well as fibrosis, resulting in heart failure (61). Data from the U.S Renal Data System suggests that HD in itself is an independent risk factor for development of *de novo* heart failure and once diagnosed, the two-year mortality is as high as 51%. Left ventricular hypertrophy is an important precursor of HF in HD patients. It results in an abnormal adaptive myocardial remodeling which is usually progressive and irreversible given that the precipitating factors are persistent in ESRD (62, 63).

The creation of arteriovenous access for HD has cardiovascular implications because of hemodynamic changes. An increase in hemodialysis AV access flow rate as well as right ventricular dilation and left atrial dilation are all risk factors associated with hemodialysis AV access induced heart failure, as are being male, having prior vascular access surgery, and having prior vascular access surgery (64). The risk of AV access induced HF appears unrelated to access type. Dialysis patients with upper arm fistula are at relatively higher risk of HF (due to increased blood flow) compared to those with forearm fistulas (64, 65). There is a temporal pattern to the pathophysiologic changes that occur in AV access induced HF (66, 67). Within days following creation of AV access, there is acute decrease in systemic vascular resistance with an increase in cardiac output through increase in stroke volume and heart rate. Peripheral vascular resistance also drops due to the access resistance phenomenon (68). As blood flow and shear stress increase, in response, the vascular endothelial cells release vasodilatory factors including nitric oxide to normalize shear stress (68). The drop in systemic vascular resistance is accompanied by a fall in systemic blood pressure. It is then the response of the sympathetic nervous system to increase stroke volume and contractility that helps maintain cardiac output in the immediate post access period (68). Persistent increase in cardiac output enhances venous to the right side of the heart which leads to right ventricular dilatation and failure (64). In the weeks to months following AV access placement, there is worsening of right ventricular dilation and dysfunction, left atrial dilatation, left ventricular hypertrophy (LVH) and myocardial remodeling (69–71). Oftentimes, left ventricular hypertrophy continues to worsen despite appropriate management of fluid and hypertension (70).

### 2.3 Coronary artery disease

Ischemic heart disease is common among patients with ESRD at the initiation of dialysis and these patients have a worse survival rate when compared to those without heart disease (72). This is understandably so as many of the factors that predispose to ischemic heart disease are already in place prior to initiating dialysis therapy (72). The estimated prevalence at the time of dialysis ranges from 15 to 73% in literature (34, 73, 74). In these patients, significant stenotic lesions of at least 75% narrowing of the reference segment abound, with majority (73%) presenting with multivessel disease and calcification. In asymptomatic patients the prevalence is as high as 53.8% (74). The likelihood of having CAD rises proportionally as the glomerular filtration rate (GFR) decreases (75). Acute myocardial infarction (AMI) is more severe and has a poorer short- and long-term prognosis in the presence of uremia (76). The proposed mechanisms of CAD include accelerated atherosclerosis by dialysis therapy and the presence of CAD risk factors prior to dialysis. Supporting this is the finding that incidence of coronary artery events among these patients does not increase in the first year, but mortality shows an upward trend (77, 78). A history of CAD adds crucial predictive information to established risk factors for deteriorating renal impairment in diabetic nephropathy (79).

There appears to be a link between coronary vascular calcification in the pathophysiology of CAD in renal disease patients (80). ESRD patients show calcific atherosclerotic disease in select vasculatures such as the coronary arteries, aorta, abdominal and lower extremity arteries (81). The pattern of calcification can be analyzed by plain radiographs showing distinct characteristics (82, 83). Here, intimal atherosclerosis and calcification exhibit a patchy distribution while medial calcification follows are pipeline-like pattern (83). Renal dialysis patients show both patterns according to studies, developing earlier relative to non ESRD patients who mainly exhibit intimal calcification pattern (80, 84–86). No existing noninvasive imaging technique can provide exact localization of intimal or medial calcification in living patients. Microscopic analysis of vascular samples from surgery or postmortem samples provides insights into this delineation. A postmortem study reviewed the relationship between the stage of kidney dysfunction and the prevalence of coronary artery calcification (80). Results showed that while intimal calcification was higher among ESRD patients, medial calcification was exclusive to this group. Patients on hemodialysis had more pronounced intimal plaque calcification attributable to uremic risk factors. Researchers noted a graded relationship between coronary artery calcium and CKD severity, even after controlling for traditional coronary artery disease risk factors in the Chronic Renal Insufficiency Cohort (CRIC) study (87). Garland et al (88) investigated the impact of coronary artery calcium in CAD development in 125 CKD patients for decline in renal function and discovered that coronary artery calcium score of 100 to 399 has 7.4 increased odds of decline in renal function, and a score >400 correlate with 8.8 increased odds of decline in kidney function at 1 year of follow up. There is a difference in clinical significance between intimal and medial calcification (89). Whereas intimal calcification tends towards plaque fragility, medial calcification seems to lead to vascular stiffness. Possessing predominantly intimal calcification confers a relatively higher risk of mortality than medial calcification (83). Parfrey et al (72) investigated the outcomes and risk factors for ischemic heart disease among a cohort of 432 consecutive dialysis patients. Patients had an echocardiogram at baseline to investigate for the presence of cardiomyopathies and left ventricular hypertrophy. The authors found that ischemic heart disease is common among dialysis patients. *De novo* ischemic heart disease was independently precipitated by risk factors including older age, hyperglycemia, hypertension, hypoalbuminemia, and underlying cardiomyopathy detected on baseline echocardiography and that mortality was due to subsequent development of congestive of heart failure. In those with *de novo* ischemic heart disease while on dialysis, shorter time to event (24 months vs 55 months in non-dialysis patients) (72) has been observed.

Hyperhomocysteinemia has been reported as a powerful risk factor for atherosclerosis through the induction of oxidative stress and arterial endothelial damage (90–92). Impaired homocysteine (hcy) metabolism and extremely elevated homocysteine levels are frequently detected in individuals with ESRD (93, 94). There is an inverse relationship between rising hcy levels and decline in renal function. The fundamental etiology of hyperhomocysteinemia in renal impairment is not completely understood but seems to be related to impaired elimination by dysfunctional kidneys. Studies have shown a positive association between high plasma homocysteine levels and vascular intimal-medial wall thickness via promoting smooth muscles cells proliferation and inhibition of endothelial cell growth (95–97).

### 2.4 Valvular heart disease

**Figure 1:**
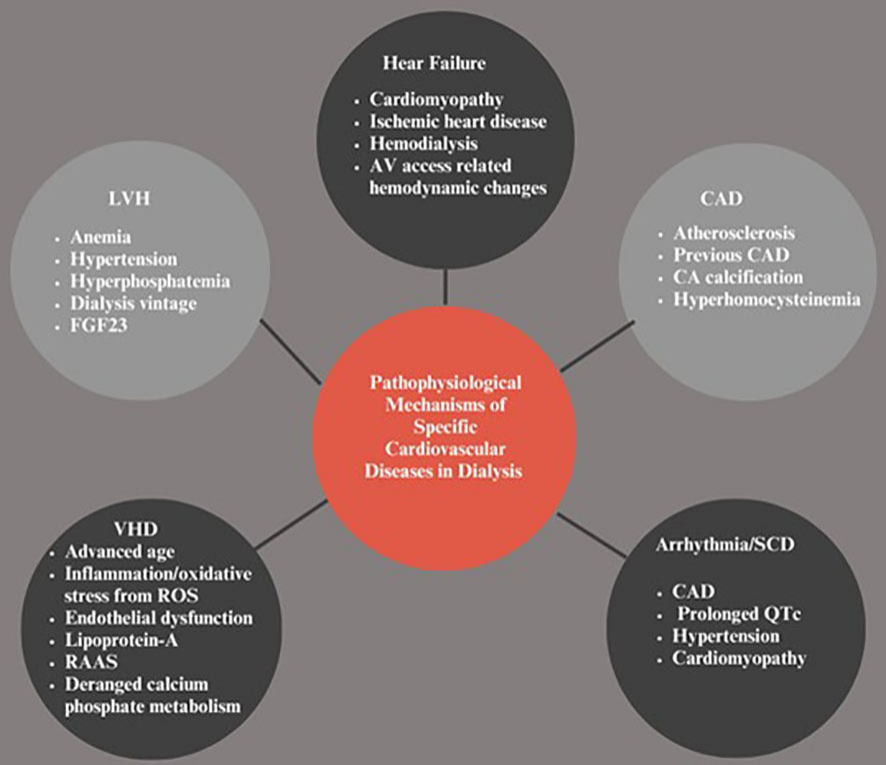
Pathophysiologic.al Mechanisms of Specific Cardiovascular Diseases in Dialysis. LVH, Left ventricular hypertrophy; VHD, Vascular heart disease; SCD, Sudden cardiac death; CAD, Coronary artery disease; CA, Coronary artery; FGF-23, Fibroblast growth factor-23; RAAS, Renin angiotensin-aldosterone system; ROS, Reactive oxygen species.

CKD patients have higher odds of left sided, aortic stenosis (AS) and mitral regurgitation (MR), not related to age or comorbidities (98). Both classic and uremia-related risk factors contribute to the elevated frequency of vascular/valvular heart disease in renal dialysis disease patients (**Figure 1**). Advanced age and dialysis vintage are principal factors associated with valvopathy. Pathological dystrophic calcification from deranged calcium-phosphate metabolism in renal dialysis involves the cardiac skeleton and valve leaflets (99, 100). Hyperphosphatemia above 6.5 mg/dL has been linked to increased mortality in ESRD patients from cardiovascular complications (101). Inflammation is thought to be a potent mediator of valvopathy. There is evidence of macrophage and T- lymphocyte deposition in diseased cardiac valves (102, 103). The pathogenesis in mitral valvopathy is via morphological destruction of the mitral valve apparatus including the leaflets, chordae, and limitation in annular motion (99, 100). A retrospective multicenter study investigating the etiological factors associated with valvular heart disease included 98 patients on chronic renal dialysis reported an annual incidence of 15-19 cases per 10,000 dialysis patients (104). The most common etiologies were endocarditis (19%) and calcific valvular heart disease (69%). Calcific valvopathy was more associated with aortic stenosis and relatively lower prevalence (9%) of multivalvular disease (>1 valvular lesion). Endocarditis induced valvopathy showed a predilection towards mitral valve involvement with more than 1 valvular lesion (32%). Aortic stenosis progresses rapidly in ESRD patients resulting in short term irreversible heart failure (104). There is an association between adynamic bone disease and valvular calcification. This is thought to be related to calcitriol and calcium containing phosphate binders (105). Valvular sclerosis predisposes to rapidly progressive valvopathy that involves the chordae tendineae, valve leaflets and cusps (106, 107). Gross inspection of the chordae tendineae reveals an uneven thickening and shortening with the histological appearance of “concentric rings of loose connective tissue surrounding a central dense core.” (107) Regurgitant and stenotic lesions are the most prevalent clinically significant valvular abnormalities identified in dialysis patients. Aortic stenosis is the most prevalent stenotic valvopathy seen in hemodialysis patients with mitral stenosis being the second most common lesion. Tricuspid and pulmonic stenosis are uncommon (108). As aforementioned, stenotic valvopathy are typically associated with annular calcification in the setting of hyperparathyroidism and an increased serum calcium-phosphorus metabolite (108). In non-dialysis patients, bicuspid aortic valve and rheumatic fever are independent risk factors in the accelerated development of aortic stenosis. However, in renal dialysis patients this senescence appears even earlier and accelerated due to uremia (108). Likewise, advanced age is an independent contributor in the development of aortic stenosis in this population (108). Histological examination of stenotic aortic valves exhibits many osteoblastic markers, including osteopontin, osteocalcin, LRP5, osteoprotegerin/RANKL/RANK, BMP-2, and Cbfa1/Runx2 (109–112). In AS, the ratio of RANKL/RANK to osteoprotegerin is skewed against osteoprotegerin, hence favoring calcifying processes (113, 114).

Endothelial dysfunction is a known contributor to development valvopathy in ESRD. Earlier in stenotic disease, there is subendothelial thickening and alteration of the basement membrane in areas of low shear stress of the valvular leaflets. The shear stress-related endothelial damage inflicted by recurrent turbulent blood flow across the valve is prevalent and pervasive in dialysis patients. In fact, fluid overload, anemia, and shunts across arteriovenous fistulae generate a state of excessive cardiac output, which results in increased flow velocity and turbulence across the valve culminating in valvopathy (102). Lipoprotein-A has been implicated in the pathogenesis of valvopathy in renal dialysis through endothelial barrier disruption, lipid deposition in the leaflets which in turn trigger local inflammation and cellular differentiation processes (115). Furthermore, angiotensin converting enzyme is found in relation to stenotic aortic valves alongside angiotensin II and apolipoprotein B. This highlights the impact of the renin angiotensin aldosterone (RAAS) system in valvopathy via extracellular matrix remodeling and fibrosis processes (116, 117). Imbalance between reactive oxygen species (superoxide and hydrogen peroxide) which are markedly elevated in calcified regions of affected vessels and low activity of the antioxidant mechanisms (superoxide dismutase enzymes) predispose to aortic valvopathy (110).

### 2.5 Arrhythmias/sudden cardiac death

Most recent epidemiological reports indicate that CVD remains the leading cause of death in CKD with arrhythmias and cardiac arrest accounting for 41.4% of all fatalities with an established cause of death (1). Lethal cardiac arrhythmia requires 3 prominent factors: triggering factors, arrhythmogenic substrates and overactive sympathetic nervous system. Prolonged QTc, hypertension and coronary artery disease are important factors in cardiac arrhythmia. According to DeLima et al. (118) hypertension induces arrhythmia through mechanical stress and inducing ischemia particularly in the context of LVH or myocardial fibrosis. In LVH, myocardial fibrotic bands prevent impulse propagation by precipitating re-entrant ventricular tachyarrhythmia (55). Prolonged QT is prevalent in dialysis patients and is thought to be due to autonomic neuropathy, cardiac myocyte hypertrophy, myocardial calcification, fibrosis and increased ventricular wall tension (119–121). Re-entry phenomenon underlies most life-threatening ventricular arrhythmias (122, 123). Altered and slow conductions are needed to propagate reentrant circuit. The aberrant regions of ventricular myocardium with delayed activation are thought to be the source of the late potential, providing the substrate for re-entry (122, 123).

Fast ventricular rate in the setting of atrial tachyarrhythmia has been hypothesized to decrease ventricular refractoriness, hence increasing susceptibility to ventricular arrhythmias (124). Moreover, atrial fibrillation (AF) can cause short-long-short sequences in the ventricular cycle length, which in turn promotes certain forms of ventricular arrhythmias (125). Overactivation of the sympathetic nervous system has both short-term and long-term proarrhythmogenic effects and contributes to the pathophysiology of arrhythmic fatalities in CKD patients. A potent mediator of sympathetic hyperstimulation is high levels of angiotensin 2 via the renin-angiotensin activation system (126, 127). Released norepinephrine stimulates the afferent renal nerves and chemoreceptors via adenosine release potentiated by MicroRNA -92 upregulation (128). Sympathetic activation influences cellular channel function, increasing repolarization heterogeneity, and contributes to cardiac fibrosis via activating -adrenergic receptors and generating inflammatory responses (129, 130). The modulation of the baroreceptor reflex has also been demonstrated to be altered in dialysis, as well as its relation to vascular calcification and arterial stiffness suggesting a potential anatomical connection between vascular alterations and baroreceptor function (131).

## 3 Roles of traditional and non-traditional risk factors in the pathogenesis of cardiovascular in end stage renal disease

**Figure 2:**
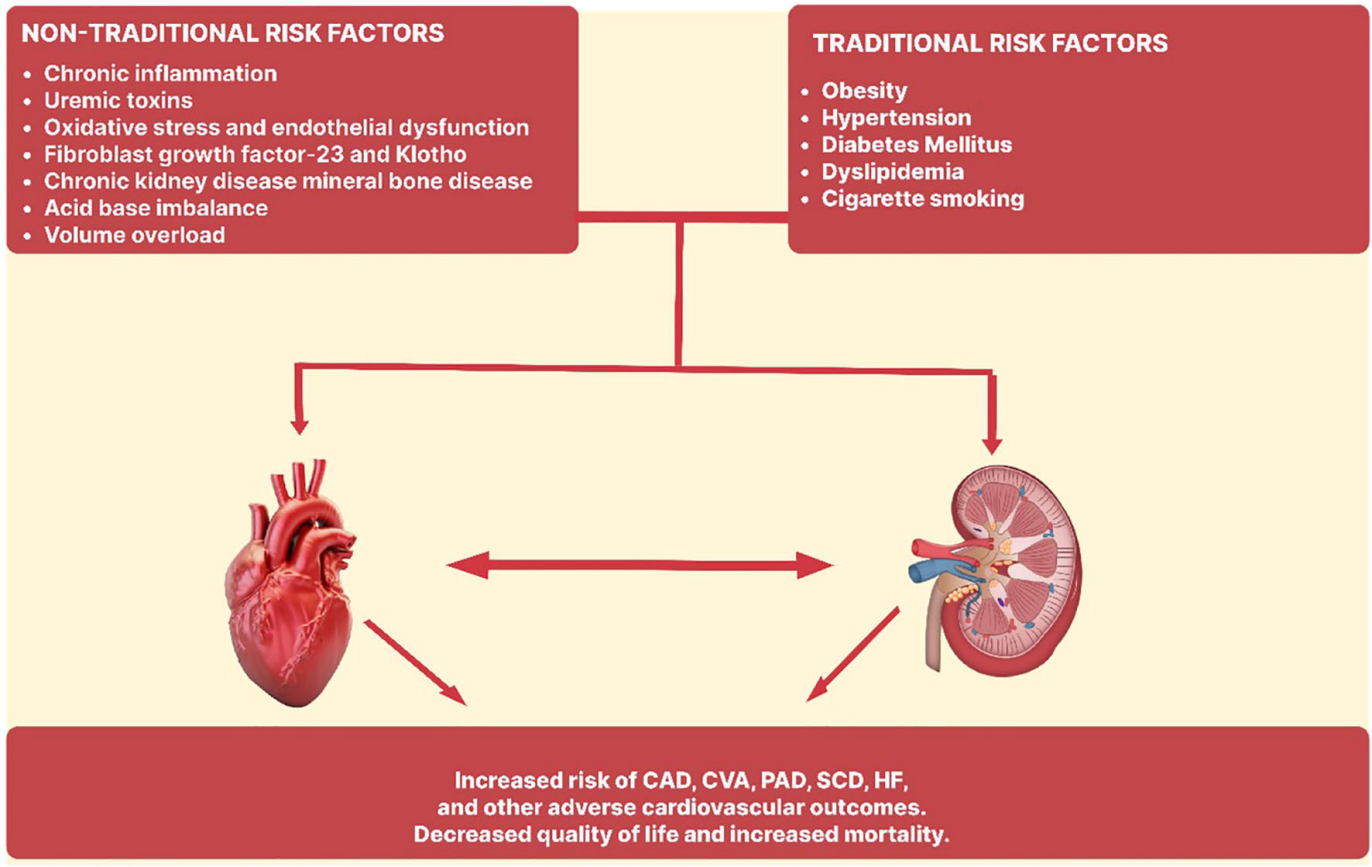
Traditional and non-traditional risk factors in the pathogenesis of cardiovascular disease in end stage renal disease. CAD, Coronary artery disease; CVA, Cerebrovascular accident; PAD, Peripheral artery disease; SCD, Sudden cardiac death; HF, Heart failure.

Cardiovascular risk assessment in dialysis patients optimizes therapeutic measures and reduces cardiovascular morbidity and death. Dialysis patients share most of the risk factors (including hypertension, diabetes mellitus, cigarette smoking, dyslipidemia, obesity) for cardiovascular morbidity and mortality in the general population (**Figure 2**). Traditional risk variables do not adequately account for the significant cardiovascular risk associated with CKD. There have been several studies exploring the roles of non-traditional risk factors such as microinflammation and vascular dysfunction, oxidative stress, uremic toxins, advanced glycation end products and volume overload. An interplay of these factors leads to the development of cardiovascular disease morbidity and mortality in this population.

## 4 Traditional risk factors

### 4.1 Diabetes mellitus

As the prevalence of diabetes mellitus (DM) rises worldwide, so does the number of individuals requiring renal replacement treatment due to complications from the disease (62, 132–134). Diabetes increases the risk of cardiovascular disease, notably acute myocardial infarction, and sudden cardiac death (135, 136). Diabetic ESRD patients have a worse 5-year adjusted survival than those with hypertension or glomerulonephritis (137). Uncontrolled DM (HbA1c > 10%) in dialysis patients increases all-cause and cardiac mortality, as well as worse 5-year survival compared to those with better control [HbA1c < 7.5%] (138–140). A target HbA1c of 7% is reasonable for glycemic control in ESRD patients with the goal of avoiding hypoglycemia. A U-shaped association between HbA1c and mortality has been demonstrated, with increased mortality in both high and low HbA1c levels (141, 142). It is important to note that there are some limitations to the use of HbA1c as an indicator of glycemic control in ESRD patients. Protein-energy malnutrition shortened red blood cell life span, anemia and the use of erythropoiesis stimulating agents have been shown to falsely reduce HbA1c. On the other hand, elevated blood urea nitrogen and metabolic acidosis are associated with falsely high HbA1c levels. Other suggested markers of glycemic control in patients with ESRD asides from HbA1c include glycated albumin and fructosamine (143).

### 4.2 Hypertension

Hypertension is a well-known risk factor for CKD. Studies do not often agree on what constitutes an adequate criterion for identifying hypertension, hence the actual prevalence may never be known with any degree of precision. In a multi-center trial of over 2000 HD patients in which hypertension was defined as the use of antihypertensive medications or weekly average pre-dialysis systolic blood pressure > 150/85 mmHg, the prevalence of hypertension was 86% (144). Similarly, a prevalence of 82% was reported in another HD cohort with the definition of hypertension being the prescription of any antihypertensive medication or inter-dialytic ambulatory blood pressure ≥ 135/85 mmHg (145). Defining blood pressure at ≥140/90 mmHg among patients on peritoneal dialysis, the prevalence of hypertension was found to be between 69 to 88% (146). Due to variations in pre-dialysis and post-dialysis BP readings, ambulatory blood pressure monitoring [ABPM] is the most reliable method to diagnose hypertension in dialysis patients and has the added advantage of being reproducible and able to provide information on circadian variation (147, 148). In comparison to dialysis unit recordings, home BP recordings, including self-measured readings and ABPM and self-measured readings are greater predictors of cardiovascular and all-cause mortality. Therapy with anti-hypertensive medications is associated with a reduction in cardiovascular morbidity and mortality in ESRD patients. A meta-analysis demonstrated that BP lowering with medications reduced the risk of cardiovascular events as well as cardiovascular and all-cause mortality in dialysis patients (149). Similarly, several studies have shown a reduction in cardiovascular morbidity; cardiovascular and all-cause mortality with the use of angiotensin receptor blockers for BP control in ESRD patients (150, 151). Despite these proven benefits, the optimal target BP has not been determined (152).

### 4.3 Obesity

In the general population, obesity is associated with increased cardiovascular and all-cause mortality; however, a paradoxically inverse association has been observed between body mass index and mortality in hemodialysis patients (153–158). This obesity paradox does not necessarily imply that the mechanisms contributing to obesity associated cardiovascular outcomes in ESRD patients is entirely different from that of the general population, but that this pathway could be obscured by other dominating factors in ESRD (159). Several explanations for the obesity paradox include inflammation and protein calorie wasting, time disparities among competing factors among others (159). A chronic inflammatory state induced by chronic kidney disease leads to proteolysis and appetite suppression in dialysis patients due to the release of cytokines such as interleukin-6 and tumor necrosis factor alpha (160, 161). It is thought that obesity may provide a ‘functional reserve’, potentially diminishing the effect of inflammation and protein energy wasting in patients with a higher body mass index (BMI). Another view is that the competing short-term effects of inflammation and protein-energy wasting (PEW) may outweigh the conventional long-term deleterious effects of obesity in dialysis patients who already have a high mortality (159). ESRD This paradoxical association is less evident in PD patients compared to HD patients. An observational study of PD patients reported lower cardiovascular and all-cause mortality in patients with high BMI/high muscle mass compared to those with normal BMI/high muscle mass. It also demonstrated an increased risk of cardiovascular and all-cause mortality in patients with a high BMI and low muscle mass (162).

### 4.4 Dyslipidemia

Dyslipidemia is an important risk factor for cardiovascular mortality in the general population with risk scores designed based on lipid levels and other risk factors to determine the need for initiation of interventions to reduce cardiovascular risk. In ESRD patients, dyslipidemia is majorly characterized by normal to low total cholesterol and low-density lipoprotein, high triglycerides, and low high-density lipoprotein (163). The relationship between cardiovascular risk and serum cholesterol levels in patients on dialysis is complex. An inverse relationship has been reported in observational studies between total cholesterol and survival in dialysis patients (164–166). However, when corrected for C-reactive protein, Interleukin-6 and serum albumin, the association between cholesterol and mortality reflects that seen in the general population; suggesting that the inverse relationship reported is probably confounded by chronic inflammation and malnutrition (167). In ESRD patients, it appears that the role of cholesterol in atherosclerosis differs; and a five-fold increase in the prevalence of vascular calcification as well as greater deposition of inflammatory cytokines and more intense intra-plaque hemorrhage have been reported in coronary artery studies (163). In a study comparing the effect of lipid lowering therapy in CKD and non-CKD patients with coronary artery disease, the carotid artery media/intima thickness reduced significantly with atorvastatin therapy during the 2-year observation period in the non-CKD group while this change was not observed in the CKD cohort and it was suggested that the beneficial effect of statins is likely negated by the uremic milieu (168). There is an accumulation of markedly atherogenic lipoproteins including lipoprotein A, oxidized low density lipoprotein (LDL), intermediate-density lipoproteins, small dense LDL and very low-density lipoprotein (VLDL) due to the deficiency of hepatic lipase, lipoprotein lipase and LDL receptor-related protein in ESRD patients (169). This atherogenic lipid profile has been reported to be more prominent in the PD population compared to patients on hemodialysis. In the 2021 European Society of Cardiology (ESC) guidelines for dyslipidemia, severe CKD, with estimated glomerular filtration rate (eGFR) of 30 mL/min/1.73 m2, and moderate CKD, with eGFR of 30–59 mL/min/1.73 m2, are categorized as very high and high cardiovascular (CV) risks, respectively. Similarly, Kidney Disease Improving Global Outcomes (KDIGO) guidelines integrates albuminuria and renal function into CVD risk estimation for CKD patients (170). A validation study in this regard was implemented on a large database with information from 1.8 million patients. The investigators found an increased prevalence of high cardiovascular risk CKD patients (<0.001) in both men and women than was previously determined based on scoring systems excluding these 2 values (171).

### 4.5 Cigarette smoking

Cigarette smoking is an important cause of preventable death in the United States with ischemic cardiovascular disease contributing a third of these deaths (172). Among dialysis patients, the reported prevalence of smoking is about 15% (173). A systematic review and meta-analysis of smoking and cardiovascular outcomes in dialysis patients showed a significant increase in all-cause mortality among smokers, although no significant increase was seen in cardiovascular events (174). These findings were thought to be due to the fact that smoking may contribute to mortality through non-cardiovascular mechanisms and that the occurrence of cardiovascular events was probably multifactorial in this group of patients and was likely not influenced by smoking alone.

## 5 Non-traditional risk factors

### 5.1 Chronic inflammation

Chronic inflammation has been recognized as an important component of CKD and plays a significantly unique role in its pathophysiology as well as the development of protein-energy wasting, cardiovascular and all-cause mortality. Factors contributing to persistent inflammation in CKD include decreased excretion and increased production of pro-inflammatory cytokines, acidic milieu, recurrent and chronic infections, oxidative stress, altered metabolism of adipose tissue, and intestinal dysbiosis. Inflammation worsens with progression of CKD and in dialysis patients, where access-related infections and complications from bioincompatible materials in the dialysis circuit, microbiological quality of the dialysate and impurities in dialysis water play an additional role (175). Several biomarkers of inflammation including IL-6, IL-1β, IL-1 receptor antagonist, CRP, TNF-α, and fibrinogen were directly associated with albuminuria and inversely associated with the measures of kidney function in the Chronic Renal Insufficiency Cohort (CRIC) study (176). However, IL-6 has been found to predict cardiovascular and all-cause mortality better than other cytokines (177, 178). Current research suggests that disorders of arginine metabolism may contribute to the elevated renal and cardiovascular risk in CKD (179, 180). Patients with mild to moderate CKD have been shown to have higher serum asymmetric dimethylarginine (ADMA) and symmetric dimethyarginine (SDMA) levels which was more perverse in those with ESRD (179, 180). Endogenous methylarginines such as ADMA and SDMA as well as other methylarginines such as L-N^G^ monomethylarginine (LNMMA) have been identified as proinflammatory elements. Serum ADMA is elevated in patients with ESRD and has been associated with endothelial dysfunction and atherosclerosis; and predicts mortality in this population. ADMA inhibits nitric oxide (NO) synthase, reducing NO bioavailability and increasing the expression of IL-6 and TNF-α. Furthermore, an increased risk for CKD progression and atherosclerotic CV events has been reported in CKD patients with elevated plasma SDMA (181, 182) It is to be noted that, most of the epidemiological studies have reported conflicting data, included mostly small sized cohorts and were not uniform in their inclusion of the endogenous methylarginines (183–186). A relatively more inclusive study of the five metabolites found that SDMA had the strongest association with cardiovascular outcomes. The full cardiovascular impact of these methylarginines warrant further studies to validate these assertions.

### 5.2 Uremic toxins

Uremic toxins have been implicated as risk factors for significant cardiovascular mortality and morbidity in the ESRD population (187). The mechanisms by which they negatively impact the cardiovascular system are incompletely understood but are thought to be multifactorial. Uremic toxins cause cardiovascular damage through their effects on leukocytes, endothelial cells, smooth muscle cells and platelets (188). Several other uremic toxins aside from urea and creatinine have been identified in ESRD patients. These have been divided into small molecules, middle molecules and protein-bound molecules based on their physicochemical properties. Small molecules include urea, creatinine, creatine, uric acid, asymmetrical dimethylarginine, symmetrical dimethylarginine among others. They generally have a molecular weight (MW) ≤ 500 Daltons, are water soluble and not bound to proteins. Middle molecules have a MW of >500 Da and are removed only by hemodialysis employing high-flux membranes with the prototype being B2-microglobulin. The protein-bound molecules generally have a low MW, many are toxic and are difficult to remove via HD. These include hippuric acid, para hydroxy hippuric acid, homocysteine, indole-3-acetic acid, indoxyl sulfate, p-Cresol and retinol binding protein (189).

Under the influence of uremic toxins, leukocytes are less responsive to activation and stimulation, resulting in infections, chronic microinflammations, malnutrition, and atherosclerosis. Uremic states lead to macrophage activation via upregulation of TNF-α and stimulation of nuclear factor-kappa B (NF-κB) which in turn drives vascular damage, including aortic calcification. via oxidative stress and high superoxide anion levels (190–192). Furthermore, they cause disruption of the endothelial glycocalyx leading to increased microvascular permeability and systemic vascular dysfunction. They also decrease tissue plasminogen factor and increase plasminogen activator inhibitor-1 and von Willebrand factor (192). Dysregulation of vascular tone, production of reactive oxygen species, inhibition of nitric oxide production and promotion of cellular senescence are reported detrimental effects of uremic toxins. Consequently, these substances increase the proliferation of vascular smooth muscle cells, resulting in thickening and calcification of the aorta (24, 193). Thrombosis is another cardiovascular manifestation of uremia (194). Patients with uremia have been reported to have enhanced caspase-3 activity which mediates exposure of phosphatidylserine on the outer membrane of platelets and these activated platelets are more prone to Thrombosis (194).

p-Cresol tends to accumulate in CKD patients due to reduced excretion and alterations in the gut microbiome. It is the product of bacterial degradation of tyrosine and phenylalanine in the gut and is absorbed from the gut as its main metabolites, p-cresylsulfate (pCS) and p-cresylglucuronide, two protein-bound small molecules. Elevated plasma concentrations of p-cresol are strongly linked to CV risk in CKD (195, 196). Indoxyl sulfate (IS), on the other hand, is an end-product of tryptophan metabolism that is albumin-bound. It accelerates the progression of CKD and stimulates oxidative stress and inflammation in a concentration dependent pattern (197, 198). IS has been implicated in hemostatic dysfunction, vascular damage, stimulation of tissue factor (TF) in vascular smooth muscle cells and an increase in pro-thrombotic risk in a TF-dependent manner specifically after vascular interventions (199–201).

### 5.3 Oxidative stress and endothelial dysfunction

Oxidative stress (OS) occurs when the antioxidant defense mechanisms of the body are overwhelmed by pro-oxidant molecules, and this has been found to be a significant driver of cardiovascular morbidity in dialysis patients. In CKD, there is increased production of molecules such as nitric oxide (NO) and reactive oxygen species (ROS) as well as deficient antioxidant defense mechanisms and insufficient clearance of oxidative products (202). Antioxidants, which can be endogenous or dietary supplements are lost during dialysis and leukocytes are activated, both of which result in production of ROS. The buildup of NO and ROS stimulates the activation of inflammatory mediators leading to oxidation and modification of nucleic acids, lipids, proteins, and carbohydrates (203).

Oxidative stress is now recognized as a risk factor for inflammation, atherosclerosis, diabetes mellitus, and CKD progression and in ESRD patients and it has been shown that those on hemodialysis have significantly increased OS compared to those on peritoneal dialysis (204, 205). The dialysis modality, biocompatibility of dialyzer membrane and dialysate, dialysis vintage and type of vascular access all play a part in worsening oxidative stress (206).

### 5.4 Fibroblast growth factor-23, klotho and CVD

Fibroblast growth factor-23 (FGF23) is a protein secreted by osteocytes and osteoclasts that is involved in phosphate metabolism alongside parathyroid hormone via interaction with FGF receptors. FGF23 regulates the excretion of phosphate and requires the presence of coreceptor a-Klotho to act. FGF23 reduces phosphorus levels by inhibiting the sodium-phosphate co-transporter at the proximal and distal tubules, inhibiting the production of 1,25-hydroxyvitamin D and by suppressing parathyroid hormone (PTH) production in the parathyroid glands, probably in a Klotho-dependent manner (207). FGF23 has been found to be elevated compared to PTH and phosphorus in the early stages of CKD [from stage 2] and alongside Klotho, may play a role in the prediction of CVD in ESRD. Some studies have reported an association between elevated plasma FGF23 concentration and an increased risk of CKD progression, cardiovascular complications, and mortality in ESRD. FGF23 plasma levels in coronary patients have also been associated with a higher risk of death and CV, even after adjustment for confounding factors. Soluble Klotho levels have been found to be reduced in patients with CKD and Klotho deficiency and FGF23 elevation are associated with poor outcomes and complications in CKD (208). They have both been recommended as biomarkers for adverse renal and extrarenal outcomes in patients with CKD. Serum Klotho was independently associated with arterial stiffness in a cross-section of patients with CKD and reduction in klotho worsens vascular calcification and uremic cardiomyopathy.

### 5.5 Chronic kidney disease mineral bone disease and CVD

Chronic kidney disease mineral bone disease (CKD-MBD) plays a profound role in the development of cardiovascular calcifications, which in turn is associated with cardiovascular morbidity and mortality in patients with CKD. The process of vascular calcification is a complex active regulated process resembling bone osteogenesis and is not just a simple deposition of calcium phosphate crystals (209). It involves a transformation of vascular smooth muscle cells into cells resembling bone progenitor cells with an upregulation of osteochondrogenesis resulting in a loss of contractile competence of the vessel as well as the production of a collagen matrix and the formation of calcium–phosphorus-rich vesicles leading to the mineralization process of the vessel internal surface (210). Cardiovascular calcification can increase the incidence of sudden cardiac death, arrhythmias, stroke and mortality in patients with ESRD; It can lead to ischemic CVD and elevated pulse pressure and pulse wave velocity resulting in a reduction of diastolic coronary perfusion with consequent development of left ventricular hypertrophy [LVH] which is a well-known CV risk factor. Aortic stenosis is a well-documented consequence of vascular calcification which results in an increase in afterload, leading to LVH (211). A meta-analysis demonstrated an increased risk of mortality with increasing severity of calcifications and there was a correlation between the number of calcified valves and the risk of mortality. Cardiovascular calcifications increase the risk of both cardiovascular and all-cause mortality; therefore, the detection of valve calcification is essential for risk stratification in ESRD patients. Computed tomography (CT) is the gold standard for the evaluation of vascular calcification, but plain radiographs are an acceptable cheaper alternative with less radiation exposure, and lateral abdominal radiographs are recommended by KDIGO to detect the presence or absence of vascular calcification in CKD Stage 3–5 patients as a reasonable alternative to CT-based imaging (212). Aside from lateral abdominal radiographs, plain radiographs of the chest, pelvis, both hands and feet can also be used to detect calcifications of the aortic arch, iliac and femoral arteries and the small arteries of the hands and feet respectively.

### 5.6 Acid-base imbalance and CVD

Acid base imbalance: majorly characterized by metabolic acidosis is common in ESRD patients due to a decreased renal production of bicarbonate and a reduction in the ability of the kidneys to excrete non-volatile acids. Metabolic acidosis has been associated with inflammation, malnutrition and increased mortality (213). In a cohort of ESRD patients on both peritoneal and hemodialysis, the adjusted risk for all-cause mortality in most subgroups with serum bicarbonate <22mEq/L was higher irrespective of the modality of dialysis and a time-averaged serum bicarbonate of <19mEq/L was associated with a higher risk of cardiovascular and all-cause mortality in peritoneal dialysis patients (213). In a cross-sectional study of 52 patients on intermittent HD that was designed to determine the association between low serum bicarbonate and cardiovascular disease; metabolic acidosis was found to be associated with diastolic dysfunction and peripheral vascular disease; although after adjusting for covariates, it was not an independent risk factor for diastolic dysfunction. They also did not find a significant association between acidosis and coronary artery disease or systolic dysfunction (214).

### 5.7 Volume overload and CVD

Volume overload is quite common in dialysis patients and has been found to be an independent predictor of cardiovascular and all-cause mortality (215, 216). Fluid overload was reported to be an independent predictor of increased cardiovascular and all-cause mortality in a cohort of over 300 continuous ambulatory peritoneal dialysis patients (217). Also, in a study of approximately 35,000 incident HD patients across 26 countries, in whom volume status was evaluated using bio-impedance spectroscopy, baseline fluid overload, and cumulative 1-year fluid overload exposure predicted an increased risk of mortality across all BP categories with the highest risk of mortality in those with fluid overload and systolic BP < 130 mmHg at baseline and at 1 year (218). There is a need to maintain the dry weight of dialysis patients with sodium and volume restriction as well as ultrafiltration during dialysis. In the bid to control volume, ultrafiltration rates should generally not exceed 13 ml/kg/h as a retrospective analysis of about 118,000 HD patients found that ultrafiltration rates > 13 ml/kg/h were associated with higher mortality compared with rates ≤ 13 ml/kg/h (219, 220).

### 5.8 Hyperhomocysteinemia

Homocysteine is a non-essential amino acid that is involved in the metabolism of methionine. In the methionine pathway, the conversion of homocysteine back to methionine is catalyzed by methyltetrahydrofolate and methylcobalamin; hence deficiencies of folate and vitamin B12 can lead to accumulation of excess homocysteine which is referred to as hyperhomocysteinemia. Hyperhomocysteinemia is thought to be implicated in atherothrombosis, vascular calcification and cardiovascular disease (221). Patients on dialysis are at risk of malnutrition as well as micronutrient deficiencies including folate and vitamin B12 and it has been reported that 85–100% of ESRD patients have hyperhomocysteinemia and it is associated with increased cardiovascular morbidity and mortality in this population (220, 222). A meta-analysis of 7 RCTs performed to assess if homocysteine-lowering with folic acid reduced cardiovascular events in patients with ESRD found a significant reduction in risk, with the greatest benefit seen in patients who had a > 20% decrease in homocysteine levels, a longer duration of therapy and no or partial folic acid fortification (223). On the other hand, another systematic review of 6 randomized control trials (RCTs) found that homocysteine-lowering therapy had little or no effect on cardiovascular or all-cause mortality in patients with ESRD (224).

## 6 Screening for cardiovascular disease in dialysis patients

### 6.1 LVH/heart failure

*De novo* and recurrent heart failure affects morbidity and mortality as well as the capacity to provide appropriate dialysis in most patients on dialysis. The KDIGO recommends echocardiographic assessment of all dialysis patients with clinical indications for heart failure or cardiomyopathy like the methods and indications in the general population (225). In fact, there is a grade B recommendation to perform echocardiogram in all patients at the initiation of dialysis following volume and electrolyte optimization, ideally within 1 to 3 months and then at 3-yearly intervals. Much like the general population, dialysis patients with *de novo* reduced LV systolic function (EF 40%) should be assessed for coronary artery disease. This may include stress testing and/or coronary angiography. However, given the reduced diagnostic accuracy of stress tests in CKD patients, coronary angiography may be preferred in individuals at high risk for CAD, such as those with diabetic CKD. This is true even for people who have had negative stress imaging tests (225). Echocardiography provides information on cardiac chamber volumes, LV mass, systolic and diastolic function. LVH is defined as having an LV mass index of more than 134 g/m2 of body surface area for men and less than 110 g/m2 for women on echo. ECG is a widely utilized initial screening test for LVH determination in the general population. However, traditional criteria for determining LVH has poor sensitivity in the assessment of dialysis patients. A prospective study investigating echocardiographic LV mass index (LVMI) and ECG-LVH criteria in patients with end-stage renal disease (ESRD) to determine the predictive relevance of ECG-LVH criteria noted a Sokolow Lyon voltage duration product and Cornell voltage duration product ECG criteria as the most predictive measures of LVH and cardiovascular mortality in HD patients (226). MRI offers an alternative means of assessing LV mass in dialysis patients. It has been proposed to perform better in this population due to the potential for echocardiogram to overestimate LV mass because of abnormal LV geometry and volume derangement associated with dialysis status (227).

### 6.2 Coronary heart disease

Dialysis patients are less likely to exhibit the characteristic signs and symptoms of coronary artery disease (CAD) and to be appropriately identified during an acute coronary syndrome (ACS) (76). Among the reasons for this atypical presentation are chronic elevated troponin levels without acute coronary syndrome, deconditioning and limited physical activity optimally triggering anginal symptoms, diabetes preventing localization of pain, and a low percentage of dialysis patients meeting the ST-T wave abnormality pattern criteria for ACS (76, 228–230). Troponin assays, including Troponins I and T are sensitive in diagnosing and predicting acute coronary events. As aforementioned, these enzymes are chronically elevated in ESRD patients due to several cardiac and noncardiac factors, limiting its specificity (231–233). Notwithstanding, KDIGO recommends the use of troponin T in the risk stratification of ACS with careful consideration of clinical context among renal dialysis patients (225).

Resting 12 lead ECG is a noninvasive means of investigating CAD. ESRD patients are likely to have nonspecific ST and T wave abnormalities at baseline making this diagnostic modality difficult to interpret with a sensitivity and specificity of 35% and 64%, respectively (234). A significant proportion of HD patients are physically unfit or have baseline ECG abnormalities precluding the use of exercise stress test. Pharmacologic stress test becomes an alternative. Coronary artery calcium score (CACS) is a noninvasive screening test that requires exposure to low radiation and no contrast. It is utilized in primary prevention strategies for risk stratifying patients requiring atherosclerotic disease interventions (235–238). Its utility is limited among ESRD patients due to diffuse and pervasive coronary artery calcification. In non ESRD patients, coronary artery calcification is only present in advanced atherosclerotic plaques but diffusely affects arterial media calcification reducing its diagnostic accuracy of CACS. CACS >0 has good negative predictive value and could be applied in the exclusion of obstructive CAD (239, 240). Similarly, the utility of coronary computed tomography angiography (CCTA) in diagnosing obstructed CAD is limited due to high calcification burden and artefacts in the absence of obstruction in ESRD patients (235). Myocardial perfusion scintigraphy (MPS) is a noninvasive nuclear imaging test for ischemic heart disease via administration of a radiotracer and pharmacological stress to assess myocardial viability and perfusion (241). A Cochrane meta-analysis comprising nine studies (582 participants) demonstrated that the pooled sensitivity and specificity for angiographic CAD were 0.74 and 0.70, respectively (242). Left ventricular hypertrophy, altered endothelial function in the absence of epicardial stenosis and triple-visible disease impair the reliability of the scan (235). MPS provides essential but imprecise predictive information on Cardiovascular events in ESRD patients not particularly attributable to its capacity to differentiate severe CAD in this cohort (235).

Percutaneous invasive coronary angiography is regarded as the gold standard for diagnosing coronary artery stenosis. It is invasive, costly and requires ionizing radiation and contrast media (235). Therefore, it should only be utilized after careful consideration of the clinical picture, risks and benefits. The integration of physiological parameters such as fractional flow reserve (FFR) to invasive coronary angiography improves diagnostic accuracy of CAD lesion severity (243). Just 2% of the participants of the study providing this evidence had ESRD limiting its application and generalizability in this group. FFR is an indicator of functional significance of stenotic lesions. Microvascular dysfunction, diffuse CAD, vascular calcification which are rife among dialysis patients may result in gross underestimation of the CAD stenosis severity (244, 245). Although the role of FFR in ESRD patients remains ill-defined, combining both anatomic and physiologic measures provide a superior assessment than either alone (235). Overall, invasive coronary angiography should be reserved for high-risk CAD patients presenting with symptoms and/or strongly positive stress test and would benefit from revascularization.

### 6.3 Valvular heart disease

Echocardiography is the gold standard for the evaluation of valvular anatomy and characterization of degree of valve stenosis and/or regurgitation in ESRD patients according to the updated 2017 kidney disease: Improving Global Outcomes (KDIGO) CKD-mineral bone disease recommendations (246, 247). In assessing valve regurgitation, echocardiography should be done post-dialysis when the patient has their “dry weight” and blood pressure is controlled (247). In addition to screening the heart valves, it is essential to assess the left ventricular morphology considering that a significant proportion of ESRD patients exhibit enlargement, with concentric hypertrophy being the main pattern (248). In CKD patients with severe AS, echocardiographic screening plays a major role in risk stratification and decision making to guide management strategies. There are several clinically relevant echocardiographic parameters such as left ventricular ejection fraction, wall thickness, mass index, estimated filling pressure (E/e’ ratio), left atrial volume index, estimated pulmonary artery systolic pressure (ePASP), and mean transaortic pressure gradient (98, 249). In surveilling patients with established valvular heart disease, it is recommended that patients with mild VHD be re-screened in 12 months interval, moderate VHD in 6-12 months and severe VHD in 3 months (246). The risk of infective endocarditis increases in dialysis patients, and transesophageal echocardiography should be performed if this is suspected (250). Despite advancement in cardiac CT and MRI techniques in the evaluation of several cardiac conditions, their roles in screening VHD especially in CKD patients remain subpar compared to echocardiogram (251). The risk of contrast induced nephropathy makes their use almost prohibitive. Cardiac CT scan quantify valvular and annular calcification and is often performed in CKD patients to evaluate the morphology and size of the aorta and annulus prior to transcatheter valve replacement (246, 250, 252). Furthermore, with cardiac CT, it is possible to determine the degree of AS in both low-flow, low-gradient severe AS with impaired ejection fraction and low-flow, low-gradient severe AS with intact ejection fraction (246, 252). The sensitivity of a chest X-ray for diagnosing VHD in ESRD patients is inadequate (250).

### 6.4 Arrhythmias

The standard 12 lead electrocardiogram (ECG) is helpful in assessing conduction abnormalities and is often the initial investigation in dialysis patient suspected of cardiac arrythmias. Notably, dialysis related fluid and electrolytes shifts may affect the ECG waveform and likely impair interpretation (253). Tezcan et al. (254) showed a 28% increase in intradialytic P wave duration, which was inversely correlated with potassium and magnesium concentrations at the termination of HD. Berta et al (255). found a correlation between hypokalemia and an increase in QRS length towards the conclusion of HD. Hypokalemia and hypocalcemia have been implicated in intradialytic QTc prolongations. ECGs should be evaluated based on the recording period with reference to HD therapy timing and the patient’s fluid and electrolyte status.

Due to fluid-related alterations in QRS amplitude, post-HD ECGs are likely to indicate left ventricular hypertrophy (253). Variations in QRS duration measurement in relation to HD may affect the evaluation of intraventricular conduction delays which could have management implications. Heart rate variability (HRV) is an indirect noninvasive method for assessing tachograms of continuous ECGs. It analyzes the cardiac autonomic modulation based on the variations in cardiac pacemaker reactivity to autonomic activations (256). Abnormal HRV parameters have been linked to cardiovascular mortality among dialysis patients (257–260). Decreased HRV in CKD patients reflects an aberrant autonomic nervous system response (261). Continuous ECG monitoring via implantable loop recorders may be obtained in dialysis patients vulnerable to substantial conduction abnormalities, including ventricular arrhythmias, or atrial fibrillation or flutter to facilitate early diagnosis and commencement of appropriate management (262). It offers remote monitoring and transmission capabilities. Sudden cardiac death is a major cause of mortality among dialysis patients. Study data from prolonged cardiac rhythm monitoring at the time of death among dialysis patients via implantable loop recorders points to bradycardia and asystole as the most common rhythms degenerating to sudden cardiac death rather than ventricular arrhythmia seen in the general population (262, 263). Echocardiography can assess for structural abnormalities such as valvular dysfunction, left atrial enlargement, and left ventricular hypertrophy, which are not uncommon in dialysis patients and could predispose to arrhythmias (264). Cardiac magnetic resonance imaging is of little prognostic utility, is cumbersome and cost prohibitive relative to various forms of ECG monitoring (264).

## 7 Use of atherosclerotic cardiovascular disease risk calculator in dialysis patients

Existing data support the use of an atherosclerotic cardiovascular disease (ASCVD) calculator to assess the presence of risk variables for future ASCVD events in asymptomatic adults without CKD (265). Age, sex, race, blood pressure, cholesterol, diabetes mellitus, smoking status, and therapy with antihypertensive drugs, statins, and aspirin are all factors considered by the ASCVD risk calculator. However, the study validation for the use of ASCVD parameters in risk classification did not include dialysis patients. Nonetheless, it is recommended in asymptomatic CKD patients without cardiovascular disease (266).

## 8 Cardiac biomarkers in dialysis

### 8.1 Cardiac troponin

Troponin I, and Troponin T are released into the circulation in the event of cardiac myocyte injury and necrosis following ischemia, inflammation, or trauma. High troponin levels are linked to higher mortality in dialysis patients even in those with preserved left ventricular function (267, 268). In non-CKD/ESRD population, elevations in troponins in conjunction with clinical symptom and electrocardiographic (ECG) changes are used to diagnose acute myocardial injury and to determine short- and long-term adverse outcomes. The high prevalence of persistently elevated troponin levels in asymptomatic ESRD patients makes it difficult to interpret cardiac troponin levels. Troponin T relative to I isotype is the most elevated of the two, therefore several assessment assays screen troponin T. It is unclear whether the chronic troponin elevation is linked to impaired kidney clearance or enhanced cardiac release. Several lines of evidence support that increased cardiac release of troponins, rather than decreased clearance, is the primary etiology of elevated basal levels in these patients (269, 270). The study by Diris et al. supports the theory of poor renal clearance (271). The authors demonstrated that troponin T, despite being a large molecule, is degraded before being excreted by the kidneys and can thus still be detected by traditional methods. Subclinical myocardial damage from micro-infarctions and those associated with left ventricular hypertrophy without clinical evidence of acute myocardial ischemia have also been proposed as mechanisms contributing to chronic troponin elevation (272–274). In renal dialysis patients, several studies have proposed different mechanisms by which hemodialysis and ESRD state could contribute to troponin elevation. These include elevated ventricular wall pressure and stretch, coronary microvascular dysfunction, anemia, hypotension, uremia, hemoconcentration, dialysis related cardiac injury, decreased clearance, or binding to dialysis membrane of cardiac troponin fragments (275).

Although there is limited supporting data, it is believed that decreased level of cardiac troponin I (cTnI) among patients on peritoneal dialysis patients compared to hemodialysis patients (276). A small study in critical care patients undergoing continuous renal replacement therapy (CRRT) revealed a decline in cardiac troponin level in the first 5 to10 hours after initiation of CRRT. Therefore, when interpreting serial cardiac troponins in dialysis patients for the diagnosis of acute myocardial infarction (AMI) timing of dialysis should be considered. Hemodialysis enhances troponin clearance therefore assays obtained in the immediate period could lead to falsely low levels. Miller-Hodges et al (233) in their study to risk stratify dialysis patients based on high sensitivity troponin found that in patients being assessed for acute coronary syndrome, high-sensitivity cardiac troponin categorized fewer individuals with renal impairment as low risk, and a majority as high risk, specificity was also reduced for type 1 MI. Several other risk stratification tools including the European Society of Cardiology 0/1-hour algorithm for rapid rule in and rule out in non-ST elevation MI, have been studied but they lacked in sensitivity, specificity, and low utility in the diagnosis of acute coronary syndromes (232, 277).

Use of serial troponin levels are preferred to diagnose acute myocardial infarction in addition to clinical symptoms and suggestive ECG findings in patients with normal kidney function as well as ESRD patients (278). There is no consensus about the level of cardiac troponin T (cTnT) or cTnI elevation needed to diagnose AMI among patients with CKD. Despite baseline elevations in troponin, it remains invaluable in diagnosing acute myocardial ischemia in the right clinical setting. Either cardiac troponin I or T may be used for diagnosis of acute MI. The Fourth Universal Definition of Myocardial Infarction recommends a change in troponin elevation above 20% from baseline or above the 99^th^ percentile of values established in healthy individuals, for the determination of ACS ESRD patients on dialysis when accompanied by clinical symptoms and suggestive ECG findings (279). A persistently elevated troponin level, with no signs of rising or falling patterns, is most likely a sign of chronic myocardial damage, even if substantial (279). Undulating values are more likely related to other types of cardiac ischemia other than type 1 and may as well be suggestive of acute volume overload or CHF.

### 8.2 B natriuretic peptide

B type Natriuretic peptide (BNP) is produced in the cardiac myocytes, secreted predominantly from the ventricles. Ventricular cells are recruited to secrete BNP in response to high ventricular filling pressure, volume overload, and left ventricular dysfunction in both symptomatic and asymptomatic subjects. BNP has diuretic, natriuretic, and hypotensive effects. It also inhibits the renin-angiotensin system, endothelin secretion, and systemic and renal sympathetic activity. Plasma BNP has a variety of clinical immunoassays, which include rapid point of care assay and central lab assays. A 100 ng/L threshold is set for these assays, but correlation is weak above or below this level. A potential source of error lie in the fact that plasma BNP concentration can vary with the assay used, age, sex, and body mass index. The normal values of BNP and N-terminal pro b-type natriuretic peptide (NT – proBNP) tend to increase with age, and in women than men. On the other hand, they are lower in obese individuals. In normal subjects, plasma concentrations of BNP and NT-proBNP are similar. However, in subjects with LV dysfunction and ESRD, plasma NT-proBNP assays are comparatively higher than BNP. While scaling varies among BNP assays, NT-proBNP assays are standardized. An NT-proBNP level > 900 pg/mL provides equivalent accuracy as a BNP level of > 100 pg/mL for diagnosis of heart failure.

Whether or not subjects have clinically diagnosed HF, Plasma BNP and NT-proBNP concentrations are elevated in ESRD patients. Decreased GFR is directly associated with persistently elevated BNP and NT- proBNP concentrations. BNP is cleared by the receptor-mediated binding and removal, neutral endopeptidase, as well as by passive excretion so, GFR is inversely related to BNP concentrations. As a result, cutoff values for plasma BNP in patients with ESKD are different from those in patients with normal renal function. Elevation in BNP levels may also result from volume expansion or LV hypertrophy commonly found in dialysis patients.

Hemodialysis has been shown to affect serum concentrations of BNP and NT-proBNP. BNP is a smaller peptide and is cleared by low flux membranes whereas NT- proBNP is only cleared by high flux membranes. Consequently, serum concentrations of BNP and NT-proBNP depend on the type of ultrafiltration used during hemodialysis. Mahmood et al (280) The effect of peritoneal dialysis on the clearance of plasma BNP and NT-proBNP remains unclear. Neither BNP nor NT-proBNP is sufficiently accurate for diagnosing HF in patients with ESRD. According to Cheng et al (281), in stable ischemic heart disease, BNP and NT-proBNP display strong and near-identical test performance in ruling out severely reduced LVEF and in prediction of all-cause mortality or heart failure despite significant effects of age, gender, and renal function on levels of both markers.

### 8.3 Suppression of tumorigenicity-2 (ST2)

Suppression of tumorigenicity-2 (ST2) belongs to the interleukin (IL)-1 receptor family and is released under conditions of myocardial and vascular strain. ST2 consists of 2 isoforms, a transmembrane ligand (ST2 ligand) and a soluble component (sST2). The ST2 system plays an essential role in mediating myocardial and vascular remodeling and fibrosis, early atherosclerosis, and hypertension. Concentrations of sST2 are not useful in diagnosing heart failure, but they have strong prognostic value in acute and chronic CHF (282). The prognostic value of ST2 is not influenced by renal function, suggesting that ST2 may well serve as a surrogate bio marker in patients with renal insufficiency.

### 8.4 Galectin-3

Galectin-3 is a macrophage lectin product that plays a role in a cascade of events leading to tissue fibrosis which is a hallmark of cardiac remodeling and heart failure. Several studies of plasma GAL-3 as a biomarker in heart failure have been published which shows that GAL-3 was independently predictive of mortality in chronic heart failure patients. Similar to findings in the general population, GAL-3 is an independent predictor of mortality in HD patients (283). Both sST2 and GAL-3 received a class II recommendation in the American College of Cardiology/American Heart Association/Heart failure Society of America HF guidelines for risk prediction in HF.

### 8.5 Beta 2-microglobulin

Although a few studies have found a link between Beta2- Macroglobulin (B2M) and the risk of cardiovascular disease (CVD) in patients undergoing dialysis, it remains poorly understood at this time. B2M is a low molecular weight protein in the HLA complex that is similar to immunoglobulins. Due to its diminutive size, it is easily filtered at the glomerulus and subsequently metabolized by the proximal tubular cells. Serum levels tend to be higher in renal dysfunction (284). B2M has been identified as an inflammation marker and is toxic to blood vessels by contributing to amyloid formation (285). Further investigations are warranted for B2M in the context of predicting CVD risk.

### 8.6 Matrix metalloproteinases

Matrix metalloproteinases (MMPs) are a family of Zn2+- and Ca2+-dependent enzymes, which are important in the resorption of extracellular matrices in both normal physiological processes and pathological states. MMPs are one of the two systems that predominate and interact to achieve homeostasis within the vessel wall. They play key roles in the pathogenesis of atherosclerosis and postangioplasty restenosis (286). Nine MMPs have been identified. In patients with CKD and diabetics, serum levels of MMP-2, MMP-8, and MMP-9 were found to be higher, being correlated with serum phosphate (MMP-2), fibroblast growth factor-23 (FGF-23), and proteinuria (MMP-8 and MMP-9), which are both associated with oxidative stress and cardiovascular health. In addition, MMP-2 has been directly linked to vascular calcification, atherosclerotic plaque rupture, and carotid intima-media thickness, making it vital to atherogenesis (287).

### 8.7 Conclusions and future perspectives

Maintenance hemodialysis patients experience anatomical and functional cardiovascular complications, in addition to the stress related to hemodialysis. Cardiovascular mortality is still pervasive despite advancements in dialysis modalities. Nontraditional risk factors related to uremia are associated with higher cardiovascular morbidity and mortality rates in dialysis patients. The identification of cardiovascular disease risk factors and end organ damage in dialysis patients contributes greatly to the reduction of cardiovascular disease morbidity and mortality. Screening modalities in non-dialysis patients are also employed in cardiovascular assessment in this population. However, interpretation must be undertaken with careful consideration of contemporary guideline recommendations. Further research into how to effectively risk stratify these patients is desperately needed to mitigate cardiovascular complications in this vulnerable demographic.

## Author contributions

GE, DK conceptualized the topic. GE, IS, SB, IB-R and DK drafted the manuscript. GE, DK, IB-R critically reviewed the manuscript.

## Glossary

**Table:**

ABPM	Ambulatory blood pressure monitoring
ACS	Acute coronary syndrome
ADMA	Asymmetric dimethylarginine
AF	Atrial fibrillation
AMI	Acute myocardial infarction
AS	Aortic stenosis
ASCVD	Atherosclerotic cardiovascular disease
AV	Arteriovenous
BNP	B-type natriuretic peptide
BP	Blood pressure
BMI	Body mass index
CACS	Coronary artery calcium score
CAD	Coronary artery disease
CCTA	Coronary computed tomography angiography
CHF	Congestive heart failure
CKD	Chronic kidney disease
CKD-MBD	Chronic kidney disease mineral bone disease
CRRT	Continuous renal replacement therapy
CRP	C-reactive protein
CRIC	Chronic Renal Insufficiency Cohort
CT	Computed tomography
cTnT	Cardiac troponin T
cTnI	Cardiac troponin I
CV	Cardiovascular
CVD	Cardiovascular disease
DM	Diabetes mellitus
ECG	Electrocardiogram
EF	Ejection fraction
ESC	European Society of Cardiology
eGFR	Estimated glomerular filtration rate
Epasp	Estimated pulmonary artery systolic pressure
EPO	Erythropoietin
ESRD	End stage renal disease
FGF-23	Fibroblast growth factor 23
FFR	Fractional flow reserve
GAL-3	Galectin-3
GFR	Glomerular filtration rate
HbA1c	Glycated hemoglobin
Hcy	Homocysteine
HD	Hemodialysis
HF	Heart failure
HRV	Heart rate variability
IL	Interleukin
IS	Indoxyl sulfate
KDIGO	Kidney Disease Improving Global Outcomes
LDL	Low density lipoprotein
LNMMA	L-N^G^ monomethylarginine
LV	Left ventricular
LVH	Left ventricular hypertrophy
MDRD	Modification of Diet in Renal Disease
MI	Myocardial infarction
MPS	Myocardial perfusion scintigraphy
MR	Mitral regurgitation
MRI	Magnetic resonance imaging
MW	Molecular weight
NT-proBNP	N-terminal pro b-type natriuretic peptide
NO	Nitric oxide
OS	Oxidative stress
pCS	p-cresylsulfate
PD	Peritoneal dialysis
PEW	Protein energy wasting
PTH	Parathyroid hormone
RAAS	Renin angiotensin aldosterone system
RCT	Randomized control trial
ROS	Reactive oxygen species
SBP	Systolic blood pressure
ST-2	Suppression of tumorigenicity-2
SDMA	Symmetric dimethylarginine
TF	Tissue factor
TNF- α	Tumor necrosis factor-alfa
USRDS	United States Renal Data System
VHD	Valvular heart disease
VLDL	Very low-density lipoprotein
